# Activation of Nrf2 signaling and suppression of oxidative stress-induced neuronal apoptosis: mechanisms for baicalin capsules relieving diabetic encephalopathy

**DOI:** 10.3389/fphar.2026.1740727

**Published:** 2026-06-26

**Authors:** Ruimin Tian, Xianfeng Zhou, Juan Pan, Chunlei Yu, Xiaolin Lian, Kejia Lei, Jieyu Liu, Yifei Liu, Hongyun Xi, Lijun Luo, Jing Wen

**Affiliations:** 1 Department of Pharmacology, North Sichuan Medical College, Nanchong, China; 2 School of Pharmacy, North Sichuan Medical College, Nanchong, China; 3 Chongqing Three Gorges Medical College, Chongqing, China; 4 School of Anesthesiology, North Sichuan Medical College, Nanchong, China

**Keywords:** apoptosis, baicalin capsules, diabetic encephalopathy, Nrf2, oxidative stress

## Abstract

**Introduction:**

Baicalin is the most abundant flavonoid in the traditional Chinese heat-clearing botanical drug *Scutellaria baicalensis* Georgi. and has been demonstrated to exert a protective role in various brain diseases. Baicalin capsules (BC), a preparation composed primarily of baicalin, have been widely used in the clinical treatment of acute bronchitis, chronic hepatitis, hypertension, cerebral ischemia sequelae, diabetic retinopathy, and other conditions. Nevertheless, the effects of BC on diabetic encephalopathy remain largely unexplored. The aim of this study was to investigate the therapeutic effects and underlying mechanisms of BC on diabetic encephalopathy.

**Methods:**

HT22 cells were treated with glucose and palmitic acid to mimic neuronal damage in type 2 diabetes mellitus (T2DM). The CCK-8 assay and oxidative stress-related detection kits were employed to measure cell viability and cellular oxidative stress levels. The ultrastructure was observed using transmission electron microscopy (TEM). The baicalin content in BC was detected through high-performance liquid chromatography. The cognitive ability was evaluated using the Morris water maze in db/db mice. Nissl staining, TEM, and TUNEL staining were performed to assess neuronal injury. The expression of Nrf2 signaling and apoptosis-related proteins was determined by western blotting and immunofluorescence colocalization analyses.

**Results:**

We successfully established an *in vitro* model of type 2 diabetic encephalopathy. *In vitro* experiments revealed that baicalin significantly enhanced the cell viability, reduced ROS production, and attenuated apoptosis. *In vivo* results demonstrated that BC improved learning and memory dysfunction in db/db mice. BC markedly increased the number of Nissl bodies, reduced ROS and MDA levels, and increased SOD and GSH-Px activities in the frontotemporal cortex of db/db mice. Furthermore, BC attenuated neuronal apoptotic characteristics, and decreased the ratio of TUNEL-positive neurons and the expression of cleaved caspase-3. Additionally, BC stimulated Nrf2 activation by increasing the expression of Nrf2, HO-1, NQO1, and GPX4 proteins, as well as promoting the colocalization of neuronal nuclei marker-NeuN with Nrf2 or GPX4.

**Conclusion:**

BC ameliorated diabetic encephalopathy in db/db mice. The underlying mechanisms were closely associated with Nrf2 activation in neurons of the frontotemporal cortex, along with the inhibition of oxidative stress-induced neuronal apoptosis.

## 1 Introduction

Based on the latest data from 2024, approximately 589 million adults (20–79 years) worldwide had diabetes and more than 90% of them were diagnosed with type 2 diabetes (IDF, 2025). Notably, the prevalence is expected to continue to rise (Młynarska et al., 2025). As a global health problem, diabetes is closely associated with varying degrees of cognitive dysfunction, ranging from mild cognitive decline to severe dementia (Moran et al., 2022). This complication of the central nervous system is termed diabetic encephalopathy (DE). Because the precise mechanism of DE remains unclear, valid preventive and therapeutic interventions have yet to be developed.

Accumulating evidence suggests that oxidative stress plays a pivotal role in the onset and progression of DE (Erukainure et al., 2019; Groeneveld et al., 2019; Mazumdar and Singh, 2024). The brain requires a substantial supply of oxygen and glucose to sustain energy production. In diabetes, chronic hyperglycemia intensifies cerebral oxidative metabolism, resulting in uncontrolled generation of reactive oxygen species (ROS) and other free radicals. This cascade subsequently triggers oxidative stress (Rossetti et al., 1990), which contributes to various forms of cell death, including apoptosis, pyroptosis, and ferroptosis, among others (Liu et al., 2025; Maiese, 2023). Several studies have confirmed that cognitive dysfunction in diabetic animal models can be ameliorated by mitigating oxidative stress and neuronal death (Dhaliwal et al., 2022; Xie et al., 2023). Nuclear factor erythroid 2–related factor 2 (Nrf2) is a transcription factor. Except for maintaining redox homeostasis by modulating the expression of a series of downstream antioxidant proteins, including heme oxygenase-1 (HO-1), NAD(P)H: quinone oxidoreductase 1 (NQO1), glutathione peroxidase 4 (GPX4), superoxide dismutase (SOD), and others (He et al., 2020; Silva-Islas and Maldonado, 2018), Nrf2 activation can directly or indirectly inhibit cell apoptosis (Wang Y. L. et al., 2024), neuroinflammation (Song Z. Y. et al., 2025), and ferroptosis (Abdalkader et al., 2018; Dodson et al., 2019) in neurodegenerative diseases. Moreover, multiple drugs have improved diabetic cognitive dysfunction by reversing or upregulating the Nrf2 signaling (Hua et al., 2026; Meng et al., 2025; Song X. Q. et al., 2025). Therefore, targeting activation of Nrf2 might be a promising strategy for suppressing oxidative stress-induced neuronal death in diabetic encephalopathy.

Baicalin (Bai) is a key flavonoid in *Scutellaria baicalensis* Georgi, a traditional Chinese medicine used to purge fire and eliminate toxins (Lan et al., 2025). It exerts a variety of pharmacological effects, such as anti-inflammatory, antioxidant, antineoplastic, antiapoptotic, antidiabetic, antibacterial, and antiviral activities (Paudel and Kim, 2020; Sharawi et al., 2024; Singh et al., 2021; Wen et al., 2023). Additionally, Bai exhibits protective effects against several brain diseases, including cerebral ischemia (Long et al., 2023), traumatic brain injury (Peng et al., 2021), epilepsy (Wu et al., 2023), brain glioma (Hu et al., 2013), and Alzheimer’s disease (Zhao et al., 2023), among others. Reports indicate that Bai alleviates multiple diseases by activating the Nrf2 signaling pathway, suggesting its potential as a therapeutic drug for DE (Bao et al., 2022; Li et al., 2022). However, little is known about its effects on DE. Baicalin capsules (BC) are a Chinese patent medicine in which Bai is the sole active ingredient. BC have been widely used in clinical practice for the treatment of acute bronchitis, chronic hepatitis, hypertension, cerebral ischemia sequelae, diabetic retinopathy, and other conditions; however, its effect on DE remains unclear. In this study, we first established an *in vitro* model of neuronal injury in type 2 diabetes by exposing HT22 cells to glucose and palmitic acid and then evaluated oxidative stress and neuronal death. After assessing the neuroprotective effects of Bai, db/db mice were employed as an animal model to explore the effects and potential mechanisms of BC on DE. The findings of this study may provide novel preventive and therapeutic strategies for DE.

## 2 Materials and methods

### 2.1 Materials

#### 2.1.1 Cells and animals

The hippocampal neuronal cells (HT22 cell line, No. JNO-02001) were obtained from Guangzhou Genio Biological Technology Co., Ltd. (Guangzhou, China). Male db/m (C57BLKS/J-lepr^db/m^) (aged 10 weeks, 20–25 g) and db/db mice (C57BLKS/J-lepr^db^/lepr^db^) (aged 10 weeks, 40–50 g) were procured from Huachuang Sino Pharmaceutical Technology Co., Ltd. (Taizhou, China). Given the potential impact of hormonal fluctuations on the behavioural performance of female mice, we exclusively used male mice in this study.

#### 2.1.2 Reagents

Baicalin capsules (approval number of the National Medical Products Administration: H20158009; batch number: 1240501) were acquired from Dongguan Jinmeiji Pharmaceutical Co., Ltd. (Dongguan, China). Baicalin (>98%, No. CHB231023) was purchased from Chengdu Chroma-Biotechnology Co., Ltd. (Chengdu, China). Glucose (No. G7021) and palmitic acid (No. P0500-10G) were obtained from Sigma-Aldrich Trading Co., Ltd. (Shanghai, China). Cell counting kit-8 (No. ZH0025) was provided by Zeheng Biotechnology Co., Ltd. (Chongqing, China). Reactive oxygen (ROS, No. S0033S), malondialdehyde (MDA, No. S0131S), SOD (No. S0101S), glutathione peroxidase (GSH-Px, No. S0056), and TUNEL apoptosis (No. C1086) assay kits were procured from Beyotime Institute of Biotechnology (Shanghai, China). Primary antibodies against Nrf2 (No. 380773) were offered by Immunoway Biotechnology Co., LTD. (California, USA). Primary antibodies against HO-1 (No. 27282-1-AP), GPX4 (No. 67763-1-Ig), and cleaved-caspase3 (No. 19677-1-AP) were supplied from Proteintech Group, Inc. (Wuhan, China). Primary antibodies against NQO1 (No. DF6437), neuron-specific nuclear protein (NeuN, No. DF6145), and beta actin (No. AF7018) were provided by Affinity Biosciences (Changzhou, China). Goat Anti-Rabbit IgG(H + L) HRP (No. GAR0072) and Goat Anti-Mouse IgG(H + L) HRP (No. GAM0072) were obtained from MultiSciences (Lianke) Biotech Co., Ltd. (Hangzhou, China).

### 2.2 Cell culture and experimental treatment

#### 2.2.1 Cell culture

HT22 cells were cultured in DMEM supplemented with 10% FBS and 1% penicillin–streptomycin at 37 °C in a humidified incubator containing 5% CO_2_ (Yang et al., 2024). When HT22 cells reached 90%–95% confluence, they were passaged at a 1:2 ratio or used for subsequent experiments.

#### 2.2.2 Effects of glucose(G) and palmitic acid (PA) on cell viability

HT22 cells were seeded into 96-well plates (5 × 10^3^ cells/well) and treated with different concentrations of G and PA (33 mM G+0.5 mM PA, 50 mM G+0.5 mM PA, 60 mM G+0.5 mM PA, and 100 mM G+0.5 mM PA) for 48 h. PA was dissolved in 50% ethanol (1:1, absolute ethanol/serum-free DMEM) to prepare a 100 mM stock solution. The PA stock solution was then sterilized by 0.22 µm membrane filters and diluted with DMEM containing 5% BSA before use. CCK-8 solution (10 μL) was added to each well 1 h before the end of the incubation. Absorbance was then tested at 450 nm by a multimode microplate reader (SpectraMax M2, Molecular Devices Co., Ltd., Shanghai, China), and cell viability was calculated using the formula: Cell viability = [(As-Ab)/(Ac-Ab)] × 100%. As, Ab, and Ac represent the absorbance of the experimental, blank, and control groups, respectively.

#### 2.2.3 Effects of G and PA on ROS production in HT22 cells

The level of ROS in cells was detected using ROS assay kit. In brief, HT22 cells were seeded into 6-well plates (3 × 10^5^ cells/well) and treated with different concentrations of G and PA for 48 h. Following treatment, cells from each group were diluted into the same concentration and reacted with 10 μM DCFH-DA for 30 min in the dark at 37 °C. The cells were then washed three times to remove excess DCFH-DA outside the cells. Finally, the cells were resuspended in DMEM and measured using a fluorescence microplate reader.

#### 2.2.4 Effects of G and PA on the ultrastructure of HT22 cells

As previously described (Tian et al., 2024), transmission electron microscopy (TEM) was used to observe the ultrastructure of HT22 cells and mitochondria to evaluate cell death. Briefly, HT22 cells were cultured in 60-mm plates (1.5 × 10^6^ cells/well) and divided into two groups: control and model. The concentrations of G and PA in the model group were screened according to the results of cell viability and ROS assays. After collecting cells, 2.5% glutaraldehyde fixing solution and 1% osmium tetroxide were used for fixation, followed by dehydration with acetone, embedding with Ep812, ultrathin sectioning and staining with uranium acetate and lead citrate. The ultrastructure was then observed using TEM (JEM-1400FLASH, Tokyo, Japan).

#### 2.2.5 Effects of baicalin on cell viability of G and PA induced HT22 cells

HT22 cells were seeded into 96-well plates (5 × 10^3^ cells/well). The cells were divided into the following groups: control, model, and baicalin (1 μM, 5 μM, 10 μM, 20 μM, 30 μM, 40 μM, 50 μM, and 60 μM). Except for the control group, all other groups were exposed to the modeling concentrations of G and PA. The intervention period of baicalin was 24 h. Cell viability was measured as described previously.

#### 2.2.6 Screening the optimal inhibitory concentration of baicalin on ROS production in G- and PA-induced HT22 cells

The range of baicalin concentrations was screened based on the results of the cell viability assay. HT22 cells were then seeded into 6-well plates (3 × 10^5^ cells/well) and grouped as follows: the control group, the model group, and the baicalin group (7.5 µM, 15 μM, and 30 μM, respectively). ROS level was measured as described above to identify the optimal concentration of baicalin for subsequent mechanism-related experiments.

#### 2.2.7 Effects of baicalin on the levels of malondialdehyde (MDA), superoxide dismutase (SOD), and glutathione peroxidase (GSH-Px) in G and PA induced HT22 cells

Based on the screened baicalin concentration for ROS inhibition, the levels of MDA, SOD, and GSH-Px were measured separately on the basis of the manufacturer’s instructions to further evaluate the antioxidative effect of baicalin.

#### 2.2.8 Observation of the effects of baicalin on the ultrastructure of HT22 cells induced by glucose and PA using TEM

As described above, TEM was used to examine the ultrastructure of HT22 cells and mitochondria to assess the effects of baicalin on cell death. The cells were divided into three groups: control, model, and baicalin.

### 2.3 Analysis of baicalin in baicalin capsules (BC) by high-performance liquid chromatography (HPLC)

The baicalin standard was accurately weighed and dissolved in chromatographic-grade methanol to prepare a 1 mg/mL baicalin stock solution, 1 mL of which was precisely measured and diluted with 20 mL of methanol to obtain a 50 μg/mL working solution. Similarly, the test solution of BC (0.2 mg/mL) was prepared using the same procedure. Both standard and test solutions were filtered through a 0.22 µm membrane filter before HPLC analysis (WAYEAL-LD3000, Anhui, China). The chromatographic conditions were set as follows: the column C18 (150 mm × 4.6 mm, 5 μm; GL Sciences-Wondasil 5020–39001, Shanghai, China), column temperature 30 °C, flow rate 1.0 mL/min and injection volume 10 μL. And the mobile phase was methanol–0.1% formic acid aqueous solution (51:49). The detection wavelength was set at 280 nm (190–800 nm; WAYEAL-DAD3260, Anhui, China) (Hu, 2024).

### 2.4 Experimental animals and procedures

#### 2.4.1 Animal feeding and administration

All mice were housed in a specific-pathogen-free animal facility at North Sichuan Medical College (SYXK, Chuan, 2023–0076) under controlled with constant conditions (temperature, 21 °C ± 1 °C; relative humidity, 60%; 12: 12 h light–dark cycle). The experimental protocol was approved by the Animal Ethics Committee of North Sichuan Medical College (Appl. No. 2025041). The treatment of animals was conducted in strict accordance with the guidelines established in China’s National Animal Welfare Law. Moreover, the welfare practices adhered to European Union/ARRIVE guidelines governing the ethical care and handling of laboratory animals. Prior to the experiments, the mice were acclimatized until they reached 12 weeks of age. Mice were randomly divided into four groups (*n* = 10) using a simple randomization method based on random number tables: control, T2DM, and BC treatment (100 mg/kg/day and 200 mg/kg/day). With the exception of the mice in the control group, which were db/m mice, the other groups used db/db mice. Treatment groups received intragastric administration with different concentrations of BC aqueous solution for 8 weeks (5 days per week) at 0.1 mL/10 g. The concentration of 200 mg/kg/day was roughly equivalent to the clinical dose of BC. Both the control and T2DM groups received the same dose of distilled water. Body weight and blood glucose levels were measured every 2 weeks.

#### 2.4.2 Morris water maze test

The Morris water maze test was performed in the eighth week of BC treatment to evaluate spatial learning and memory functions of the mice. The test lasted 6 days. During the first 4 days, mice were trained to locate a hidden underwater platform. On the fifth day, a navigation positioning test was conducted, in which the escape latency was recorded within 90 s. On the sixth day, the platform was removed, and the time spent in the target quadrant and the number of times the platform area was crossed within 90 s were recorded. Mice behavior was tracked using a Morris water maze video analysis system (Techman-WMT1005, Chengdu, China). Behavioral tests were conducted by personnel not involved in the experimental design to minimize potential bias.

#### 2.4.3 Specimen collection and storage

Brain tissue samples were collected immediately after euthanasia by decapitation at the end of the Morris water maze test. The left hemispheres of three randomly selected mice from each group were isolated and fixed in 4% paraformaldehyde for Nissl staining, TUNEL staining, and immunofluorescence. The frontotemporal cortex regions of the right hemispheres were cut into 1-mm^3^ size samples and fixed in 2.5% glutaraldehyde at 4 °C for TEM observation. The remaining cortical tissue was frozen in liquid nitrogen and stored at −80 °C for western blotting analysis and biochemical assays using commercial kits.

#### 2.4.4 Nissl staining

To assess neuronal changes in the frontotemporal cortex region, brain tissues were fixed in 4% paraformaldehyde for more than 24 h, embedded in paraffin, and sectioned into 5 μm slices using a microtome (Leica-RM2235, Wetzlar, Germany). The sections were then dewaxed, stained, dehydrated, hyalinized, and sealed in turn. Finally, images were observed and analyzed under a microscope (Nikon-Eclipse E100, Tokyo, Japan).

#### 2.4.5 Measurement of ROS, MDA, SOD, and GSH-Px

A single-cell suspension was prepared using the enzymatic digestion method for the measurement of ROS (Jiancheng-E004-1-1, Nanjing, China). In detail, brain tissue from the frontotemporal cortex was collected and placed in pre-cooled PBS to wash away blood and other contaminants. The tissue was carefully minced into small pieces of about 1 mm^3^ using a ophthalmic scissor. It was subsequently incubated in a digestion solution at 37 °C for 30 min, with gentle agitation every 5 min. After terminating the digestion process using PBS, the solution was filtered through a 300-mesh nylon sieve to eliminate tissue aggregates. The cells were collected and then resuspended in PBS. Brain tissue from the same region was homogenized in lysis buffer at a ratio of 1:9 and centrifuged in a high-speed refrigerated centrifuge to obtain the supernatant, which was used for the subsequent detection of MDA, SOD, and GSH-Px. All assays were performed in accordance with the manufacturer’s instructions.

#### 2.4.6 TEM

The ultrastructure of neurons and mitochondria in the frontal and temporal lobes of mice was observed using TEM to evaluate the effects of baicalin on neuronal death. As described in our previous protocol (Tian et al., 2022), 1 mm^3^ brain tissues were washed, dehydrated, embedded, solidified, and then sliced into 60-nm-thick sections after fixation with 2.5% glutaraldehyde at 4 °C. The sections were stained sequentially with uranyl acetate and lead citrate and examined under a TEM (JEM-1400FLASH, Tokyo, Japan).

#### 2.4.7 TUNEL staining

Paraffin sections of mouse brain tissue were prepared as described for Nissl staining. After dewaxing by dimethylbenzene and gradient ethanol, and repairing by proteinase K working solution, each section was incubated with 100 μL TDT equilibration buffer for 30 min and 50 μL labeled working solution for 1 h in the dark at 37 °C respectively. The sections were counterstained with DAPI stain for 10 min at room temperature in the dark. Apoptotic neuronal cells were observed under a digital slide scanner (3DHISTECH Kft-Pannoramic SCAN II, Budapest, Hungary). Three non-overlapping visual fields within the frontotemporal cortex were selected and quantified using ImageJ software. Apoptosis rate (%) = (Number of apoptotic neurons/Total number of neurons) × 100%. All analyses were performed in a blinded manner.

#### 2.4.8 Western blotting analysis

Total protein of the frontotemporal cortical tissue samples was prepared using RIPA buffer supplemented with PMSF, and the protein concentration was detected using a BCA protein assay kit. Equal amounts of protein from each sample were separated by SDS-PAGE before transferring to PVDF membranes and blocking in 5% skim milk at room temperature for 1 h. Subsequently, the membranes were incubated with primary antibodies against Nrf2 (1:1000), HO-1 (1:1000), NQO1 (1:1000), GPX4 (1:1500), cleaved-caspase-3 (1:1000), and β-actin (1:1000) in TBST containing 5% skim milk overnight at 4 °C. After 2 h incubation with the corresponding secondary antibodies (1:10,000) at room temperature, the membranes were reacted with ECL luminescent solution, followed by images captured with a chemiluminescence instrument (ChemiScope 6100, Shanghai, China). Finally, the relative expression of each protein was quantified by ImageJ software (National Institutes of Health, Bethesda, MD, United States).

#### 2.4.9 Immunofluorescence (IF) assay

IF staining was performed as previously reported (Tian et al., 2022). Briefly, frontotemporal cortex sections were dewaxed, repaired, and incubated with 3% hydrogen peroxide solution in the dark for 15 min to block the endogenous peroxidase. After blocking with 5% goat serum for 30 min, the sections were incubated with primary antibodies against Nrf2, GPX4, and NeuN in a dilution of 1:200 overnight at 4 °C. These sections were then incubated with the corresponding secondary antibody for 40 min at room temperature in the dark, and IF images were taken using a digital slide scanner.

### 2.5 Statistical analysis

Statistical analyses were conducted using GraphPad Prism 9.5.0 version (GraphPad Software Inc., San Diego, CA, USA). Data were expressed as mean ± standard deviation (SD). Statistical significance was performed by one-way ANOVA or two-way ANOVA tests followed by Tukey’s or Dunnett’s analysis. A *P* value <0.05 was considered statistically significant.

## 3 Results

### 3.1 Effects of glucose (G) and palmitic acid (PA) on cell viability, ROS production, and ultrastructure of HT22 cells

**FIGURE 1:**
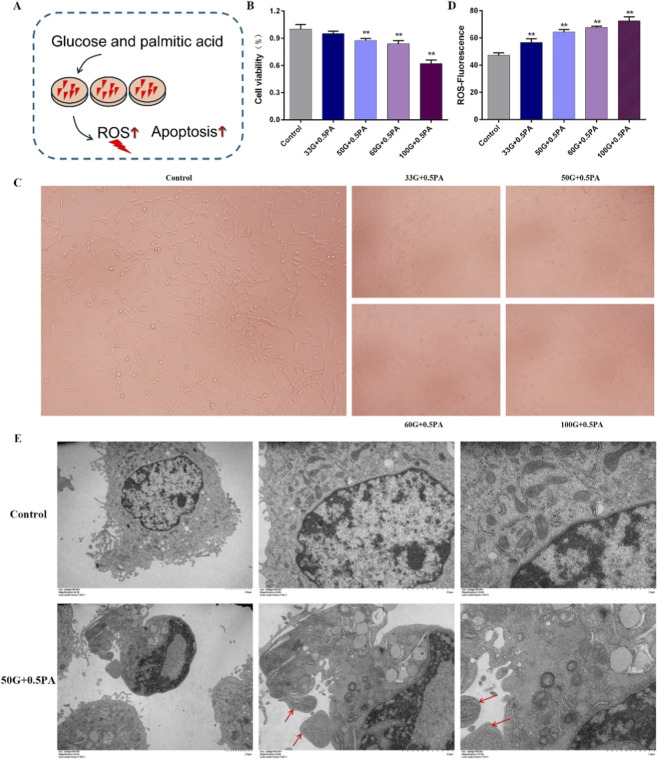
The effects of G and PA on cell viability, ROS production, and ultrastructure of HT22 cells. **(A)** The pathological mechanism diagram illustrates the influence of G and PA on ROS and apoptosis in HT22 cells. **(B)** The cell viability of HT22 cells under different G and PA insults (*n* = 6). **(C)** The characteristic morphology of HT22 cells under different G and PA insults. **(D)** The ROS production of HT22 cells under different G and PA insults (*n* = 6). **(E)** The ultrastructure of cells and mitochondria observed by TEM (scale bars for the figures in the first column are 2 μm, transmission electron microscopy [TEM] × 4000; scale bars for the figures in the second column are 2 μm, TEM × 8000; scale bars for the figures in the third column are 1 μm, TEM × 15,000; the red arrows indicate apoptotic bodies). Error bars denote SD. **P < 0.01, compared to the control group (one-way ANOVA followed by Dunnett’s *post hoc* test). G: glucose; PA: palmitic acid.

To evaluate the effects of G and PA with different concentrations on cell damage, HT22 cells were exposed for 48 h. As shown in Figure 1B, cell viability decreased in a concentration-dependent manner, with a significant reduction at 50 mM G+0.5 mM PA (*P* < 0.0001; SD = 0.022). Similarly, as the concentration of the modeling drug increased, the intercellular spaces progressively expanded, while the cells gradually adopted a rounded morphology (Figure 1C). Figure 1D shows that ROS production increased progressively with higher concentrations of G, and varied markedly from 33 mM G+0.5 mM PA to 100 mM G+0.5 mM PA (*P* < 0.0001; SD = 2.748,2.088,0.990,2.942). Additionally, ultrastructural changes of cells in Figure 1E revealed cell atrophy, chromatic condensation, apoptotic body formation, plasma membrane rupture, and cytoskeleton disintegration in the 50 mM G+0.5 mM PA group compared with the control group. Therefore, the concentration of 50 mM G+0.5 mM PA was selected as the optimal condition to mimic HT22 cell damage in the high-glucose and high-lipid environment of T2DM (Figure 1A).

### 3.2 Protective effects of baicalin (Bai) on HT22 cell injury induced by G and PA

**FIGURE 2:**
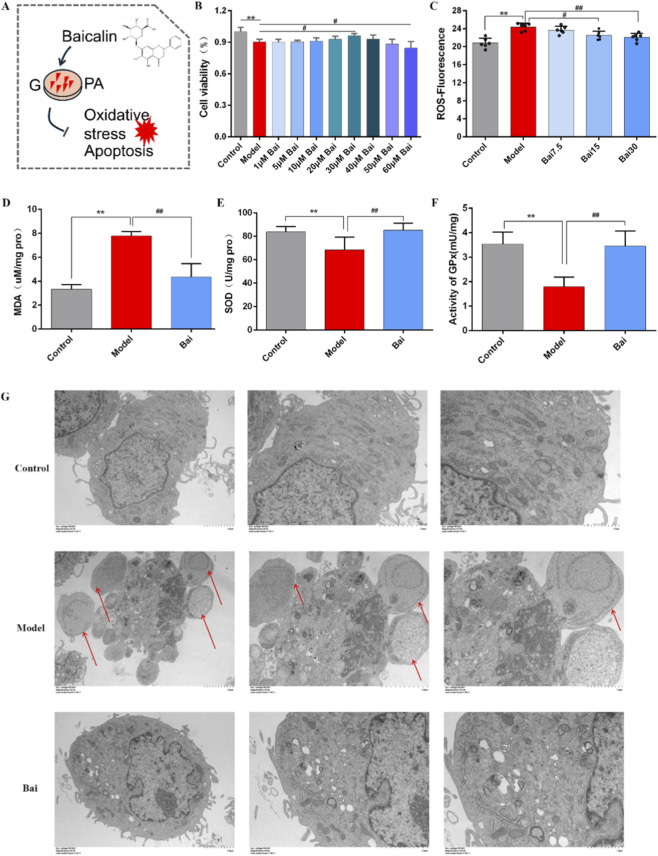
The protective effects of Bai on G- and PA-induced HT22 cell injury. **(A)** The mechanistic diagram illustrates the modulation of Bai on G- and PA-induced-oxidative stress and cell death in HT22 cells. **(B)** The effects of Bai with different concentrations on cell viability of HT22 cells induced by G and PA (*n* = 6). **(C)** The effects of Bai on the ROS level in G- and PA-induced HT22 cells (*n* = 6). The quantitative analysis of the cellular MDA content **(D)**, SOD activity **(E)**, and GPx activity **(F)** (*n* = 6). **(G)** The effects of Bai on the ultrastructure of HT22 cells induced by G and PA (scale bars for the figures in the first column are 2 μm, TEM × 5000; scale bars for the figures in the second column are 2 μm, TEM × 8000; scale bars for the figures in the third column are 1 μm, TEM × 12,000; the red arrows indicate apoptotic bodies). Error bars denote SD. **P < 0.01, compared to the control group; ^#^ *P* < 0.05 and ^##^ *P* < 0.01, compared to the model group (one-way ANOVA followed by Dunnett’s or Tukey’s *post hoc* test). Bai: baicalin.

To investigate whether Bai could attenuate HT22 cell injury induced by G and PA, a CCK-8 assay was performed. As shown in Figure 2B, compared with the model group, cell viability increased in a concentration-dependent manner ranging from 1 μM to 30 μM of Bai treatment, peaking at 30 μM (*P* = 0.0373; SD = 0.019). In Figure 2C, Bai at15 μM (*P* = 0.0131; SD = 0.940) and 30 μM (*P* = 0.0020; SD = 0.870) significantly reduced ROS production, with the greatest inhibition at 30 μM. Furthermore, the content of MDA, and the activities of SOD and GPx were apparently reversed by 30 μM of Bai in Figures 2D–F. Moreover, almost no apoptotic features were observed in the cells of the 30 μM Bai intervention compared with the model group (Figure 2G). These results indicate that baicalin mitigates the HT22 cell damage induced by G and PA, and its protective effects are associated with antioxidant and antiapoptotic mechanisms (Figure 2A).

### 3.3 HPLC analysis of baicalin capsule (BC)

**FIGURE 3:**
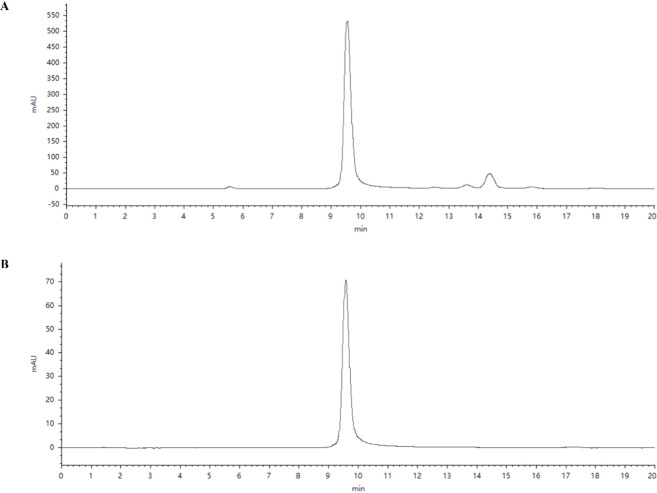
The HPLC of BC **(A)** and baicalin standard **(B)**. HPLC: High-performance liquid chromatography; BC: baicalin capsule.

The baicalin content in BC was determined using HPLC analysis. The chromatographic profiles of the BC sample and baicalin standard are shown in Figures 3A,B respectively. The baseline noise at a wavelength of 280 nm was minimal and did not significantly influence the baicalin detection. The retention time of baicalin was approximately 9.692 min, with the peak fully separated from other impurity peaks. The relative amount of baicalin was 31.05 mg/capsule (124.17 mg/g BC). These findings suggest that baicalin is the primary constituent of BC.

### 3.4 Effects of BC on body weight, blood glucose, spatial learning-related behaviors, and brain histomorphology in db/db mice

**FIGURE 4:**
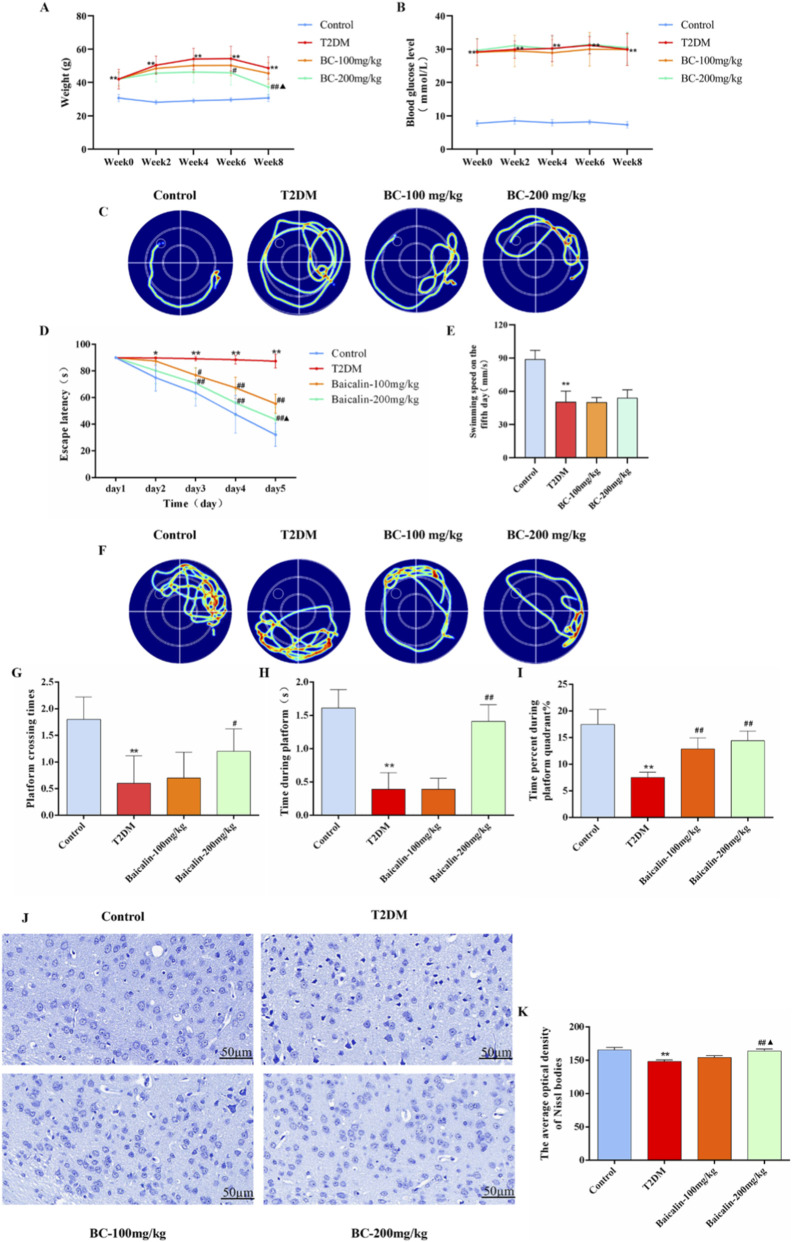
Effects of BC on body weight, blood glucose level, spatial learning-related behaviors, and brain histomorphological characteristics in db/db mice. **(A)** Body weight of mice in each group during 8 weeks of BC treatment (*n* = 10). **(B)** Blood glucose levels of mice in each group during 8 weeks of BC treatment (*n* = 10). **(C)** The swimming paths of the fifth training day. **(D)** The escape latency of five continuous days (*n* = 10). **(E)** The swimming speed on the fifth day (*n* = 10). **(F)** The swimming paths of the probe trial. The platform crossing times **(G)**, time during platform **(H)**, and time percent during platform quadrant **(I)** in the probe trial (*n* = 10). The Nissl-stained neurons **(J)** and statistical results **(K)** of the frontal temporal cortex (*n* = 3). Error bars denote SD. *P < 0.05 and **P < 0.01, compared to the control group; ^#^ *P* < 0.05 and ^##^ *P* < 0.01, compared to the T2DM group; ^▲^ *P* < 0.05, compared to the BC-100 mg/kg group (one-way ANOVA or two-way ANOVA followed by Dunnett’s or Tukey’s *post hoc* test).

During the experimental period, db/db mice consistently demonstrated significantly higher body weight and blood glucose levels than db/m mice (Figures 4A,B), consistent with previous findings of this diabetic animal model. Body weight in db/db mice was significantly reduced beginning in the sixth week of 200 mg/kg BC treatment. However, no significant alterations in blood glucose levels were observed after BC treatment. In the place navigation test, escape latency of db/db mice was significantly prolonged from the second day to the fifth day compared to the control group. Conversely, treatment with 100 mg/kg and 200 mg/kg of BC markedly shortened the escape latency from the third day to the fifth day (Figures 4C,D). The swimming speed of db/db mice on the fifth day was significantly reduced in comparison with control group (*P* < 0.0001; SD = 9.500). However, compared with mice in T2DM group, no significant change was observed in the speed of mice across the different BC groups (Figure 4E). The spatial probe test in Figures 4F–I showed that the platform crossing times, time during platform, and time percent during platform quadrant of db/db mice were apparently less than those in the control group; conversely, treatment with 200 mg/kg of BC could significantly increase the platform crossing times (*P* = 0.0307; SD = 0.421) and time during platform (*P* < 0.0001; SD = 0.251) of db/db mice, and the time percent during platform quadrant of db/db mice in the two BC treatment groups was remarkably increased (*P* < 0.0001; SD = 2.116,1.825). As shown in Figures 4J,K, abundant Nissl bodies were observed in the frontotemporal cortex of the control group, whereas their number was markedly decreased in the db/db mice, and 200 mg/kg of BC improved these lesions significantly (*P* = 0.0009; SD = 2.932). These findings indicate that BC ameliorate spatial learning-related behaviors and brain histomorphological abnormalities in db/db mice. Notably, these beneficial effects may occur independent of blood glucose control. Moreover, a 200 mg/kg dosage of BC demonstrated superior efficacy, supporting its selection for subsequent studies.

### 3.5 Effects of BC on oxidative stress and apoptotic injury in the frontotemporal cortex of db/db mice

**FIGURE 5:**
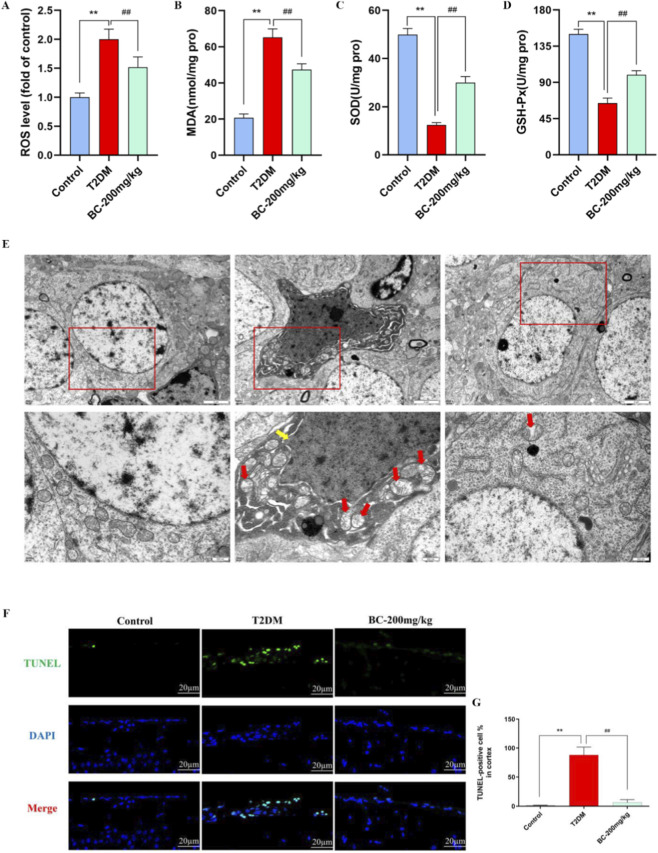
Effects of BC on the oxidative stress and apoptosis injury in the frontotemporal cortex of db/db mice. The quantitative analysis of the cellular ROS level **(A)**, MDA content **(B)**, SOD activity **(C)**, and GSH-Px activity **(D)** (*n* = 6). **(E)** The ultrastructure of neuron and mitochondria (scale bars for the upper figures are 2 μm, TEM × 8000; scale bars for the lower figures are 500 nm, TEM × 20,000). The yellow arrow indicates nuclear condensation. The red arrows indicate mitochondrial swelling and pronounced loss of cristae. **(F)** The representative images of TUNEL staining. **(G)** The quantitative analysis of TUNEL positive cell rate (*n* = 3). Error bars denote SD. **P < 0.01, compared to the control group; ^##^ *P* < 0.01, compared to the T2DM group (one-way ANOVA followed by Dunnett’s *post hoc* test).

**FIGURE 6:**
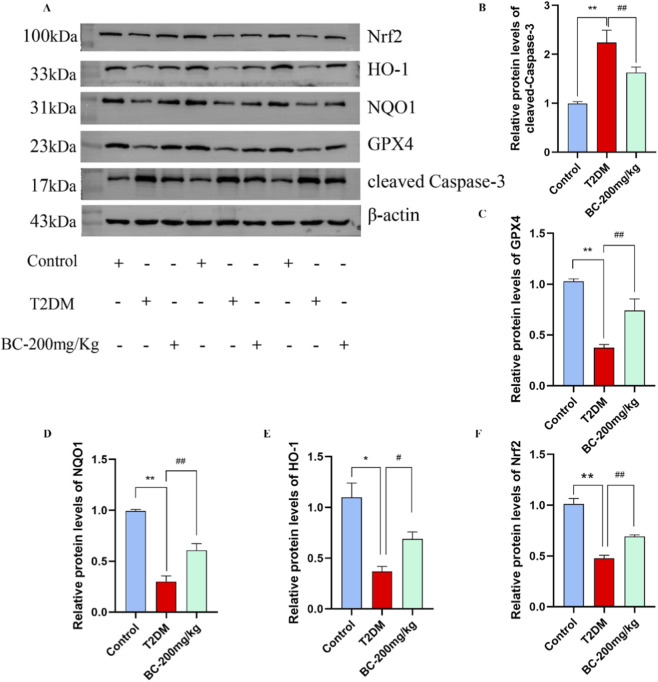
Effects of BC on the expression of apoptosis and Nrf2 activation-related proteins in the frontotemporal cortex of db/db mice. **(A)** The representative bands of Nrf2, HO-1, NQO1, GPX4, and cleaved Caspase-3. The corresponding quantification of cleaved Caspase-3 **(B)**, GPX4 **(C)**, NQO1 **(D)**, HO-1 **(E)**, and Nrf2 **(F)** (*n* = 3). Error bars denote SD. *P < 0.05 and **P < 0.01, compared to the control group; ^#^ *P* < 0.05 and ^##^ *P* < 0.01, compared to the T2DM group (one-way ANOVA followed by Dunnett’s *post hoc* test).

ROS and MDA levels were significantly elevated in the frontotemporal cortex of db/db mice, while the activities of SOD and GSH-Px were markedly decreased. Treatment with 200 mg/kg of BC therapy significantly reversed these changes (*P* = 0.0001; SD = 2.476 or *P* < 0.0001; SD = 5.065) (Figures 5A–D). TEM analysis (Figure 5E) revealed distinct apoptotic features in neurons of db/db mice, including cellular atrophy, nuclear condensation, mitochondrial swelling, and pronounced loss of cristae. In contrast, following BC treatment, almost no apoptosis was detected in neurons, whereas an only mild reduction in mitochondrial cristae was observed. Consistently, TUNEL staining indicated that the ratio of TUNEL-positive neurons surged in db/db mice, which was significantly reduced after BC treatment (Figures 5F,G). Moreover, the expression of cleaved caspase-3 was elevated in db/db mice (*P* = 0.0002; SD = 0.256) but was downregulated following BC treatment (*P* = 0.0068; SD = 0.111) (Figures 6A,B). Collectively, these findings confirm that BC exert antioxidant and antiapoptotic effects to attenuate diabetic encephalopathy.

### 3.6 BC regulate Nrf2 activation in the frontotemporal cortex of db/db mice

**FIGURE 7:**
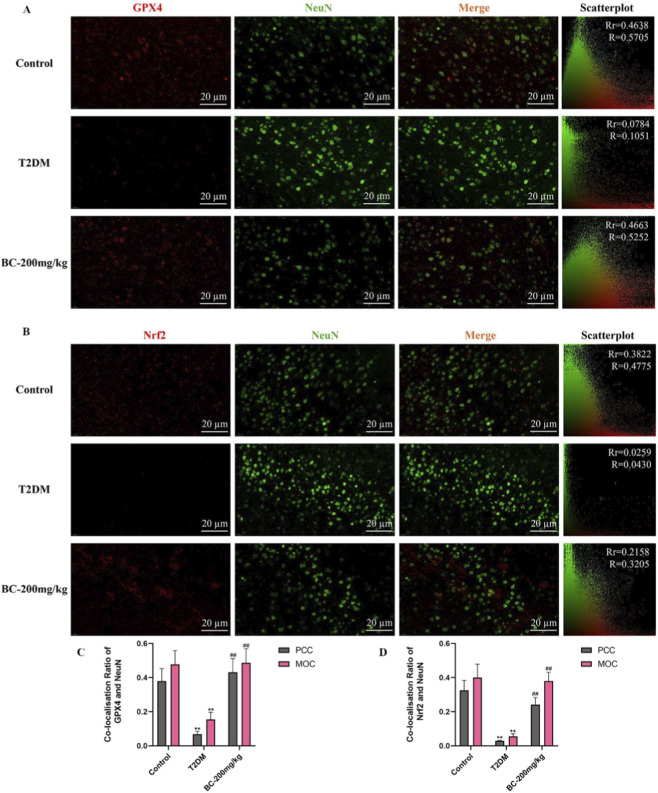
Effects of BC on Nrf2 activation in the frontotemporal cortex of db/db mice. **(A,C)** The colocalization of GPX4 and neuron by co-staining of GPX4 (red) with NeuN (green) (*n* = 3). **(B,D)** The colocalization of Nrf2 and neuron by co-staining of Nrf2 (red) with NeuN (green) (*n* = 3). Error bars denote SD. *P < 0.05 and **P < 0.01, compared to the control group; ^#^ *P* < 0.05 and ^##^ *P* < 0.01, compared to the T2DM group (two-way ANOVA followed by Tukey’s *post hoc* test). Neu: NeuN. PCC: Pearson correlation coefficient; MOC: Manders overlap coefficient.

To evaluate Nrf2 activation in the frontotemporal cortex of db/db mice, we examined the expression of critical Nrf2-related proteins through western blotting and immunofluorescent colocalization. Compared with the control group, statistical analysis showed significantly decreased levels of GPX4 (*P* < 0.0001; SD = 0.0321, Figure 6C), NQO1 (*P* < 0.0001; SD = 0.005, Figure 6D), HO-1 (*P* = 0.0002; SD = 0.050, Figure 6E), and Nrf2 (*P* < 0.0001; SD = 0.030, Figure 6F) in db/db mice. Conversely, treatment with BC significantly increased the levels of GPX4 (*P* = 0.0013; SD = 0.115, Figure 6C), NQO1 (*P* = 0.0018; SD = 0.076, Figure 6D), HO-1 (*P* = 0.0121; SD = 0.070, Figure 6E), and Nrf2 (*P* = 0.0008; SD = 0.017, Figure 6F), indicating that BC induced the activation of Nrf2. Similarly, the colocalization results revealed that BC therapy enhanced the colocalization of neuronal nuclei marker-NeuN and GPX4, as well as NeuN and Nrf2 (Figures 7A–D). These findings demonstrate that BC promote the activation of Nrf2 and increase the expression of antioxidant-related proteins in the frontotemporal cortex of db/db mice.

## 4 Discussion

DE is a severe central nervous system complication associated with diabetes, primarily characterized by cognitive impairment (Cukierman-Yaffe et al., 2020; Ding et al., 2024). Patients with DE have a higher risk of developing dementia (Chatterjee et al., 2016; Kwon et al., 2015). The commonly applied treatment is a comprehensive strategy targeting both metabolic disturbance and neurological manifestations. However, specific targeted treatment remains lacking. The development of novel agents therefore requires a deeper understanding of the underlying pathophysiology of DE.

Hyperglycemia, a hallmark of diabetes, is widely considered a primary trigger of DE. Prolonged hyperglycemia induces glucose toxicity and diminishes antioxidant defenses, resulting in oxidative stress and subsequent neuronal death (McNeilly et al., 2023; Nagayach et al., 2024). Additionally, free fatty acids represent another biomarker for diabetes. Elevated palmitic acid levels may originate from an increased synthesis of complex lipids, and this elevation triggers oxidative stress and leads to neurodegeneration through disrupting autophagy, lysosomal function, and mitochondrial function (Bergman and Ader, 2000). These effects are particularly evident in neurons because of their high metabolic demands (Saipuljumri et al., 2026). On the basis of these factors, we exposed HT22 cells to high concentrations of glucose and palmitic acid for 48 h to establish an *in vitro* model of DE. The 50 mM glucose and 0.5 mM palmitic acid significantly decreased the cell viability and increased the ROS production, indicating the occurrence of cell damage and oxidative stress. Accumulating evidence demonstrates that neuronal apoptosis triggered by oxidative stress plays a critical role in neurodegenerative diseases (Ienco et al., 2011). Under physiological conditions, the production and elimination of ROS in the body typically maintain a dynamic balance. However, this equilibrium can be disrupted when exposed to certain stimuli. Excessive ROS then begins to attack the body, triggering a series of chain reactions and subsequent neuronal apoptosis (Contestabile, 2001). Oxidative stress can initiate apoptosis via multiple pathways, including mainly the activation of NF-κB, the DNA damage and subsequent accumulation of P53, and the activation of MAPK signaling, among others (Dutta et al., 2006; Han et al., 2025; Tian et al., 2020). It was suggestive that oxidative stress-induced apoptosis may act as a culprit in DE. The TEM results in this study further revealed typical apoptotic features in HT22 cells, without evidence of other form of cell death. These findings are consistent with previous reports, confirming the successful establishment of our cell model (Ma et al., 2015; Nagayach et al., 2024; Wang et al., 2025; Yan et al., 2025).

Our previous research demonstrated that Huang-Lian-Jie-Du decoction, a traditional prescription containing baicalin, improved learning and memory performance as well as alleviated pathological brain damage in type 2 diabetic rats induced by a high-sugar, high-fat diet combined with a low dose of streptozotocin (Tian et al., 2022). However, the role of baicalin in DE remains unclear. In the present study, we investigated the effects of baicalin on HT22 cell injury induced by glucose and palmitic acid under *in vitro* conditions. Baicalin not only enhanced HT22 cell survival, but also attenuated oxidative stress and inhibited apoptosis. Ma et al. demonstrated that baicalin was capable of enhancing the behavioral performance of Wistar rats induced by streptozotocin (Ma et al., 2015). Song et al. also found that baicalin could improve the learning and memory abilities and Alzheimer's disease-related pathological features in diabetic rats induced by streptozotocin and a high-fat diet (Song Z. Y. et al., 2025). Another recent study further confirmed the neuroprotective effects of baicalin in both db/db mice and high glucose and high fat-induced GK rats (Zheng et al., 2024). These findings suggest that baicalin has promising therapeutic potential in DE. Clinically, there is a Chinese patent medicine named BC, with baicalin as its main component. Our HPLC results have confirmed this. BC have been widely used for various diseases in clinic. If BC exert therapeutic effects on DE, it would greatly benefit human health. Subsequently, we employed db/db mice as an *in vivo* model for type 2 diabetes and subjected them to 8 weeks of BC treatment. Intriguingly, BC markedly improved the learning and memory performance of db/db mice. Moreover, BC increased the abundance of Nissl bodies in the frontal temporal cortex. Because treatment with 200 mg/kg of BC (12. 417 mg/kg baicalin) produced stronger effects than 100 mg/kg dose (24.834 mg/kg baicalin), subsequent mechanistic experiments were conducted using 200 mg/kg of BC. Mechanistic results showed a marked reduction in oxidative stress and a significant decrease in the rate of TUNEL-positive cells within this zone. Additionally, expression of cleaved caspase-3 (apoptotic protein) was substantially downregulated. Furthermore, blood glucose levels in db/db mice remained stable after BC treatment, suggesting that the beneficial effects of BC on DE might be independent of glycemic regulation. Despite these findings, the precise mechanisms through which BC regulate oxidative stress and apoptosis remain incompletely understood.

**FIGURE 8:**
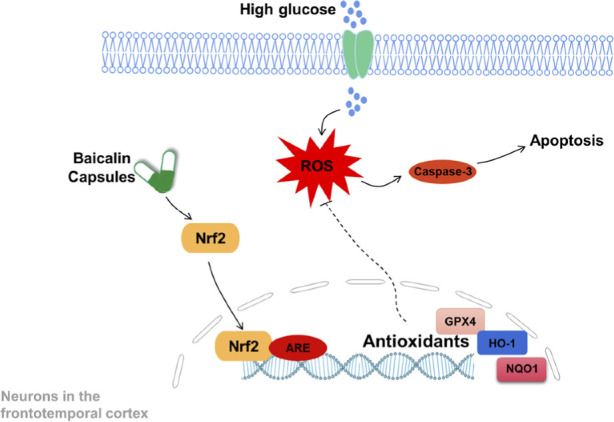
The mechanism of BC against DE: BC significantly promoted the activation of Nrf2, increased the expression of antioxidant-related proteins, and suppressed oxidative stress-induced apoptosis in the frontotemporal cortex region of db/db mice.

Nrf2 is a transcription factor that plays a critical role in maintaining redox homeostasis (He et al., 2020). Under physiological conditions, Nrf2 is predominantly localized in the cytoplasm through its interaction with Kelch-like-ECH-associated protein 1 (KEAP1), which serves as a molecular sensor of oxidative stress. Under oxidative stress conditions, Nrf2 dissociates from KEAP1, translocates into the nucleus, and promotes the transcription of antioxidant genes (Canning et al., 2015). An increasing number of studies have demonstrated that activation of Nrf2 attenuates DE. For example, Chatterjee et al. discovered that Vitamin K_2_ reversed DE partially via activating Nrf2 signaling and reducing oxidative stress and apoptosis (Chatterjee et al., 2024). Similarly, Wang et al. reported that sulforaphane ameliorated diabetes-induced cognitive dysfunction by activating the Nrf2/HO-1 pathway and reducing oxidative stress and apoptosis (Wang G. Y. et al., 2024). Therefore, targeting Nrf2 may represent a promising therapeutic strategy for DE (Cheng et al., 2024; Yuan et al., 2023). Previous evidence has also shown that baicalin prevents a variety of diseases by activating Nrf2. Consistent with this, our results revealed that BC (baicalin-containing preparations) significantly upregulated the protein expression levels of Nrf2, HO-1, NQO1, and GPX4 in the frontotemporal cortex. Moreover, increased nuclei expression of Nrf2 was confirmed by the colocalization of Nrf2 with NeuN, indicating the activation of Nrf2 by BC. In summary, our findings demonstrate that BC mitigate spatial learning-related behavioral deficits in db/db mice, which is partially associated with Nrf2 activation in the neurons of the frontotemporal cortex and the suppression of oxidative stress-induced apoptosis (Figure 8).

Nevertheless, this study has several limitations. First, an Nrf2 inhibitor was not employed to further confirm that the mechanism of BC in db/db mice was related to Nrf2. So causal validation is required in future research. Second, TEM was performed in both *in vitro* and *in vivo* experiments to evaluate apoptosis. The results have provided direct evidence for apoptosis. We further conducted TUNEL staining and WB experiments to prove apoptosis *in vivo*. However, due to the high cost of detection and limited samples, our TEM results remain descriptive. Third, no visual platform test or other vision controls were conducted, thus the influence of the animals’ visual functions on the results cannot be completely excluded and the specificity of spatial memory deficits needs additional confirmation. Besides, unlike our findings in the frontotemporal cortex, several Nrf2 activators have been shown to improve oxidative-stress and apoptosis in the hippocampus through Nrf2 activation, which may be attributed to the use of different animal models of DE (Chatterjee et al., 2024). Even in the same db/db mouse model, several Nrf2 activators can inhibit inflammation or ferroptosis via activating Nrf2 signaling (Shi et al., 2023; Song X. Q. et al., 2025). However, whether the beneficial effects of BC on DE are related to the mitigation of inflammation and ferroptosis still needs further investigation.

## 5 Conclusion

Our study revealed the phenomenon as follows: (1) An *in vitro* neuronal injury of diabetic encephalopathy was established using high concentrations of glucose and palmitic acid. This model is applicable for the high-throughput screening of novel drugs against diabetic encephalopathy. (2) Baicalin enhanced the survival of glucose- and palmitic acid-induced neuronal cells through anti-oxidative stress and anti-apoptosis. (3) Baicalin capsules have been found to exert neuroprotective effects on DE. The underlying mechanism may be associated with the activation of Nrf2 signaling in the neurons of the frontotemporal cortex, as well as the suppression of oxidative stress-induced apoptosis.

## Data Availability

The raw data supporting the conclusions of this article will be made available by the authors, without undue reservation.
